# Supplementary material for: In Silico Screening of Metal-Organic Frameworks for Formaldehyde Capture with and without Humidity by Molecular Simulation
Source: Int J Mol Sci. 2022 Nov 8;23(22):13672. doi: 10.3390/ijms232213672 (PMC9690669; doi:10.3390/ijms232213672)
Supplement: Supplementary file 1 [file ijms-23-13672-s001.zip › ijms-1991229-supplementary.pdf]

In Silico Screening of CoRE Metal-organic Frameworks for Formaldehyde Capture with or without Humidity by Molecular Simulation

Wei Li[1,*], Tiangui Liang[1], YuanChuang Lin[1], Weixiong Wu[1], Song Li[2,*]

[1]Renewable Energy Science & Engineering Institute, International Energy School, Jinan University, 519070, China

[2]State Key Laboratory of Coal Combustion, School of Energy and Power Engineering, Huazhong University of Science and Technology, 430074, China

Table S1. The force field parameters of HCHO, H_2O, N_2, and O_2

molecule	interaction site	σ/Å	(ε/k_B)/K	q/e
	C	3.75	52.9	0.45
HCHO	O	2.96	105.8	-0.45
	H	2.42	7.6	0.00
	O	3.154	78	0
H_2O	H	0	0	0.52
	M	0	0	-1.04
N_2	N	3.31	36	-0.482
	N_com	0	0	0.964
O_2	O	3.02	49	-0.112
	O_com	0	0	0.224

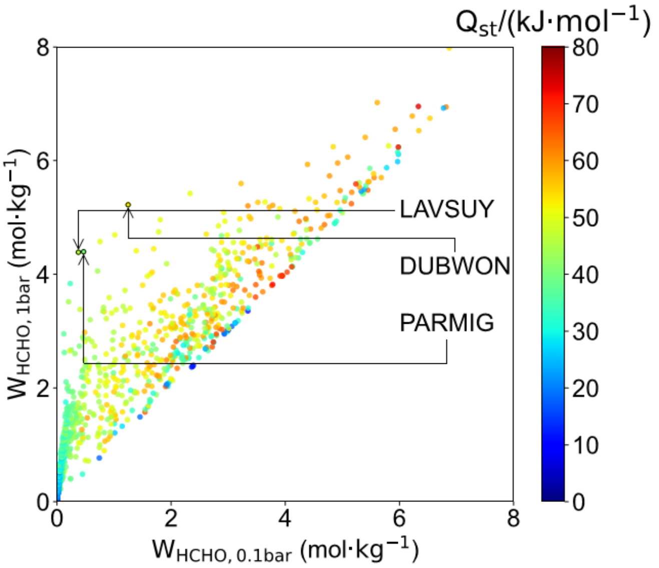

Figure S1. Relationship between HCHO capacity at 1bar and 0.1 bar, colored by heat of desorption.

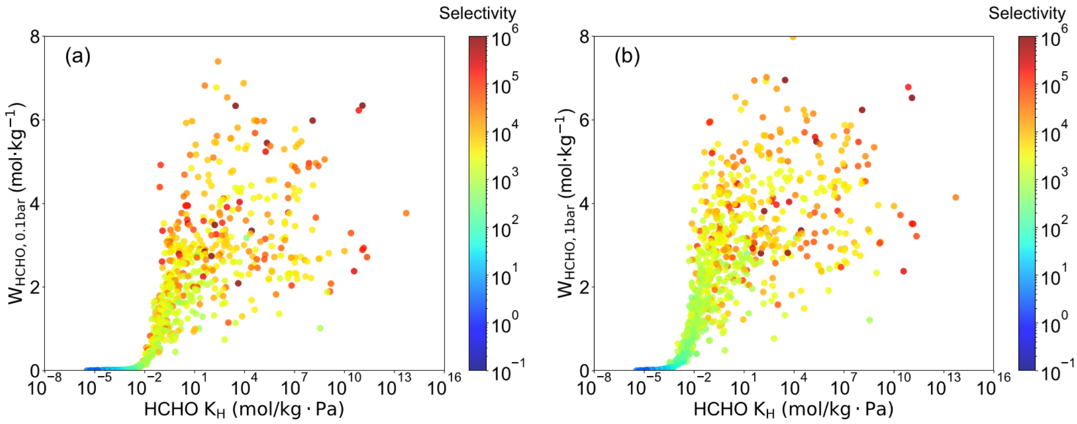

Figure S2. Relationship between HCHO K_H and (a) capactiy at 0.1 bar, 298 K, (b) capacity at 1 bar, 298 K, colored by selectivity.

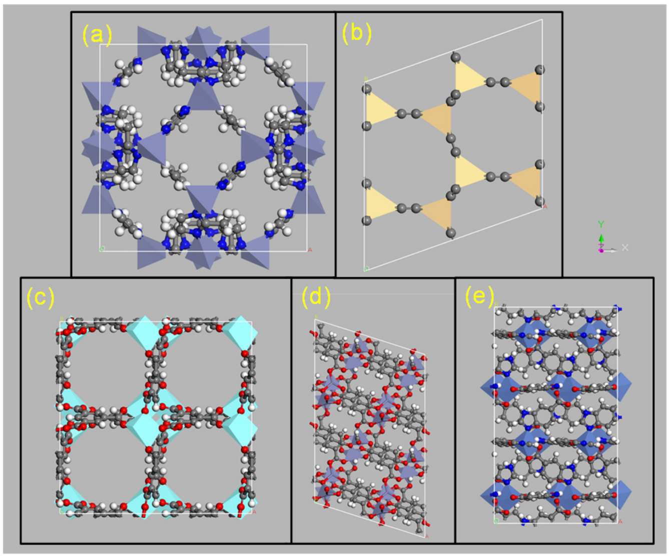

Figure S3. The snapshots of cyrstal for (a)SEHTAB , (b) DEYJIC, (c) LAVSUY (d) DUBWON, and (e)PARMIG.

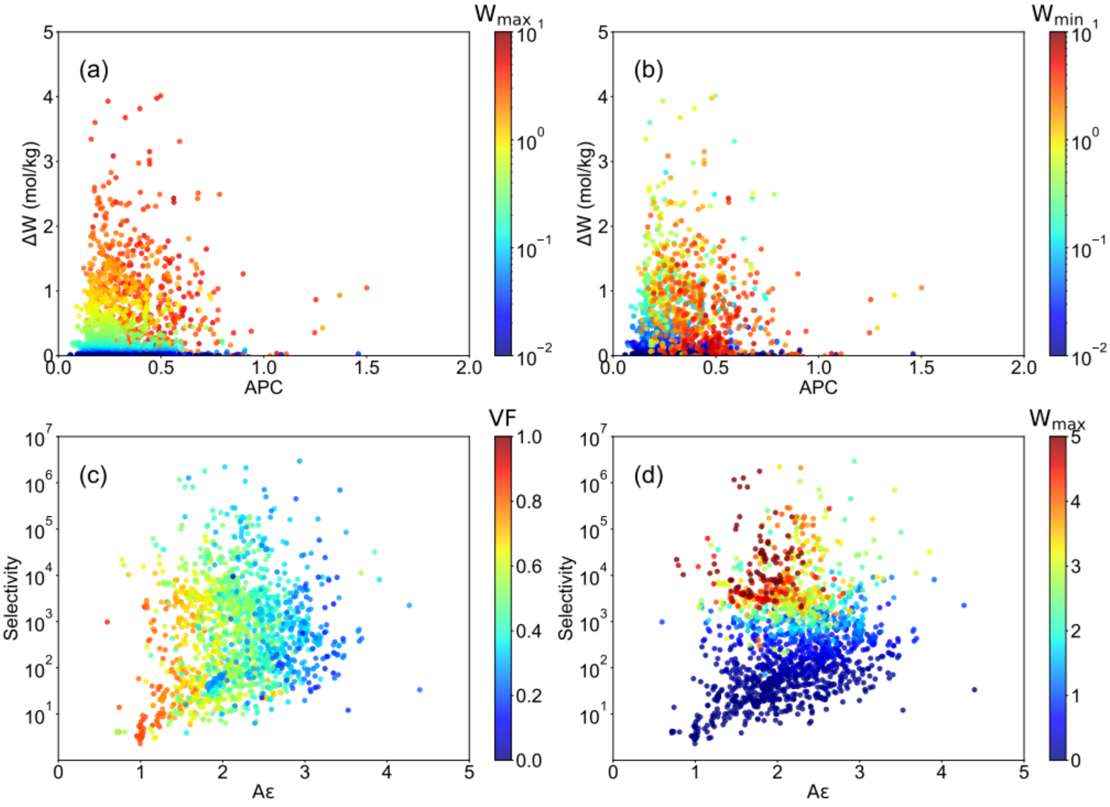

Figure S4. The relationship between chemical descriptors and formaldehyde capture performance and selectivity in 1bar, 298 K. The correlation between ΔW and APC, (a)colored by Wmax, (b) colored by W_{min} . The correlation between selectivity and Aε, (c) colored by VF, (d) colored by W_{max}.

Table S2. The void fraction(VF), minimum explanied ratio(MinER), average negative charge(ANC), average σ(Aσ) and heat of adsorption(Q_{st}) of top 10 MOFs.

REFCODE	VF	MinER	ANC<br>e	Aσ<br>Å	Q_{st}<br>kJ/mol
LAVSUY	6.62	0.19	-0.55	1.96	46.37
DUBWON	5.20	0.31	-0.48	1.93	52.18
PARMIG	4.71	0.31	-0.28	2.80	40.03
SEHTAB	5.17	0.21	-0.43	1.85	50.20
DEYJIC	4.95	0.14	-0.46	1.95	51.06
ADIQEL	4.25	0.31	-0.34	2.71	34.32
LIFWOO	4.98	0.27	-0.26	2.76	43.77
DEFKUU	5.42	0.21	-0.63	2.29	44.17
NABMUA01	6.10	0.21	-0.57	2.29	50.02
LOBHAM	6.51	0.28	-0.27	1.93	50.62

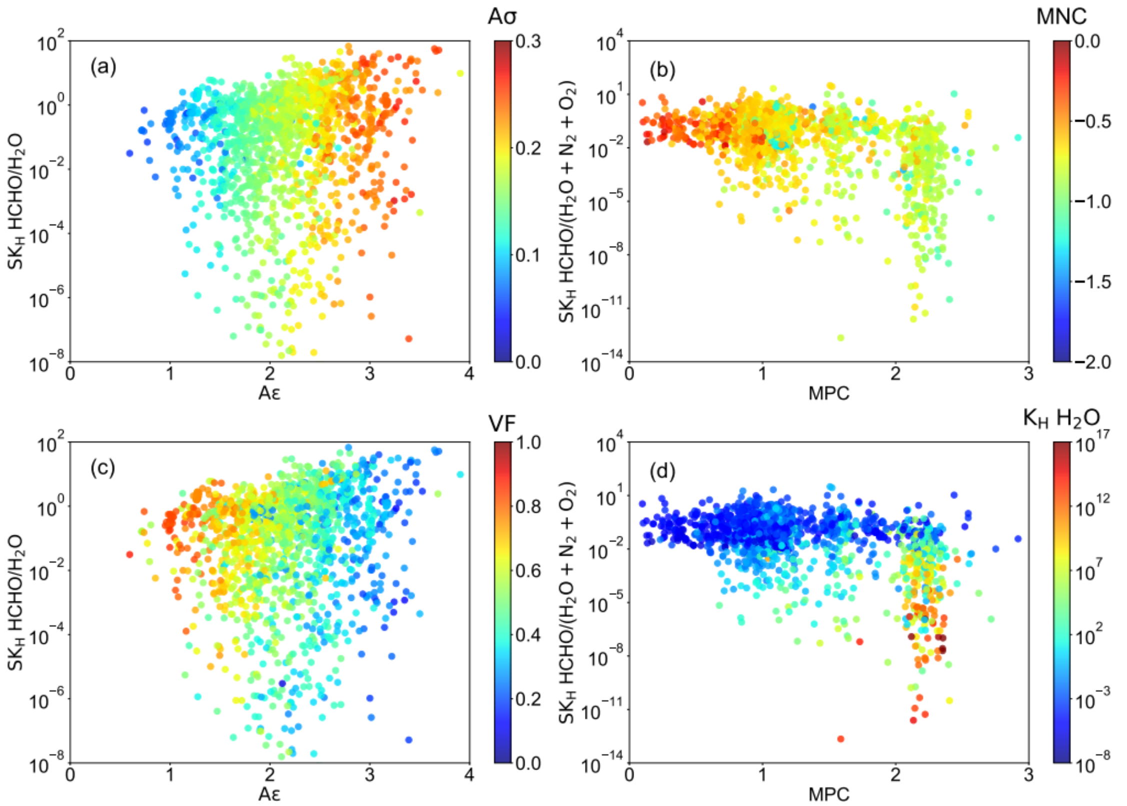

Figure S5. The correlation between henry constant selectivity HCHO/H_2O and Aε, (a)colored by the Aσ; (c) colored by the VF. The relationship between MPC and henry constant selectivity HCHO/(H_2O + N_2 + O_2), (b) colored by the MNC; (d) colored by the KH H_2O.The henry constant selectivity is calculated in a mixture component HCHO/H_2O/N_2/O_2 = 200/3280/77216/19304 at 298 K.

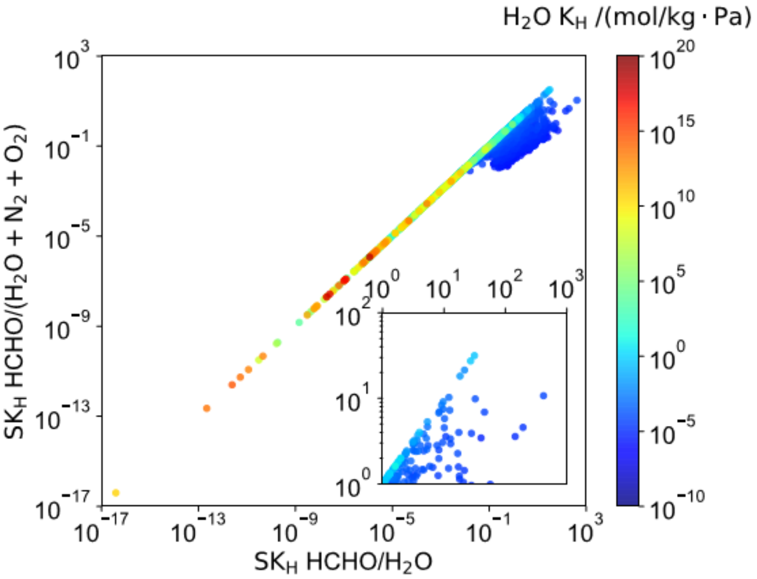

Figure S6. The relationship between between SK_H HCHO/H_2O and SK_H HCHO/(H_2O + N_2 + O_2), colored by the K_H of H_2O.
